# ASAS-NANP SYMPOSIUM: Mathematical modeling in animal nutrition—modeling in the agriculture 4.0 era and beyond: merging mechanistic knowledge and artificial intelligence with hybrid modeling

**DOI:** 10.1093/jas/skag311

**Published:** 2026-09-26

**Authors:** Jennifer L Ellis, Jessica Bode, Maureen Sahar, Jihao You, Dan Tulpan, Noud Aldenhoven, Luis O Tedeschi

**Affiliations:** Centre for Nutrition Modelling, Department of Animal Biosciences, University of Guelph, Guelph, ON N1G 2W1, Canada; Centre for Nutrition Modelling, Department of Animal Biosciences, University of Guelph, Guelph, ON N1G 2W1, Canada; Centre for Nutrition Modelling, Department of Animal Biosciences, University of Guelph, Guelph, ON N1G 2W1, Canada; Centre for Nutrition Modelling, Department of Animal Biosciences, University of Guelph, Guelph, ON N1G 2W1, Canada; Centre for Nutrition Modelling, Department of Animal Biosciences, University of Guelph, Guelph, ON N1G 2W1, Canada; Global Nutrition, Formulation & Modelling, Nutreco, Amersfoort 3811 MH, The Netherlands; Department of Animal Science, Texas A&M University, College Station, TX 77843-2471, United States

**Keywords:** mechanistic modeling, machine learning, hybrid modeling, agriculture 4.0, precision nutrition

## Abstract

With the arrival of the 4th Agricultural Revolution (Agriculture 4.0) and the influsion of animal production systems with sensors, automation, and advanced analytics, new opportunities have arisen to improve sustainability, efficiency, and the precision with which we feed animals. It is possible that the barriers of the past such as lack of automation, efficient means of data capture, and appropriate analytical tools with which to drive decision support and system optimization will be mitigated in our lifetime. Increasingly, we will see the blending of mechanistic understanding with technological advancement to guide decision-making. The emerging frontier of hybrid models representing frameworks that integrate mechanistic understanding with the pattern recognition of machine learning, offers considerable promise for application in animal systems. As an illustrative case study, research conducted in other fields where modeling approaches have been successfully integrated will be explored (for example, bioprocess engineering, weather, human health), as well as recent research in agriculture. Several architectures for hybrid modeling may be considered for application in animal biosciences: 1) Serial hybridization, where a machine learning (ML) model provides inputs into a white box model (could be mechanistic or empirical), or vice versa, 2) Parallel hybridization, where models act in parallel and ML and white box model outputs are aggregated or weighted to enhance model performance, 3) Physical constraint hybridization, where domain knowledge (such as conservation laws) typically embedded in mechanistic models are integrated into the structure, parameters or outputs of a ML model, and 4) mechanistic learning, where ML model(s) are embedded within a mechanistic model to predict specific pools or fluxes. Key challenges including data quality and integration, model interpretability, and computational efficiency are discussed alongside practical strategies to overcome them. This review examines how hybrid models can bridge the gap between data-driven insights and biological causality to support real-time decision-making in feed formulation, emissions management, and health monitoring. By positioning hybrid modeling as a cornerstone of precision livestock farming, Agriculture 4.0, and beyond, this review outlines a vision for next-generation tools that are scientifically grounded, causally robust, and prepared to support digital decision-making.

## Teaser

Automation and advanced analytics provide new opportunities to improve sustainability, efficiency, and the precision with which we feed animals. Hybrid models that combine biological knowledge with artificial intelligence offer a pathway beyond the “*data versus theory*” debate in modeling livestock systems. The integration of mechanistic understanding with machine learning approaches will enable more accurate, interpretable, and real-time decision support for Agriculture 4.0 and beyond.

## Introduction

The digital transformation of agriculture, commonly referred to as Agriculture 4.0, has the potential to reshape livestock production as we know it. Sensor technologies, genomics, and big data streams now permeate production systems, providing opportunities to enhance sustainability, efficiency, and animal welfare (Berckmans 2017; Wolfert et al. 2017; Morota et al. 2018). However, the major challenge lies in connecting and leveraging heterogeneous data sources to generate actionable insights for nutrition, health, and management. Within animal systems modeling, two dominant paradigms have emerged: mechanistic/process-based models, and pure data-driven artificial intelligence (**AI**), including machine learning (**ML**) approaches.

Classic mechanistic models (**MM**), are built from scientific first principles that govern biological systems, and are composed of algebraic, ordinary differential, or partial differential equations representing the fluxes entering or leaving pools or compartments that represent biologically meaningful aspects of the system being represented (Dijkstra et al. 1992; Gerrits et al. 1997; France and Kebreab 2008). Their integration provides a detailed accounting of flows through the system, and can be used to predict outcomes in animal biosciences such as digestibility, methane emissions, milk/egg/meat production and nutrient excretion, but also to study system behavior and test hypotheses of system regulation (France and Kebreab 2008; Baker et al. 2018). Genome-scale metabolic models (**GEMs**) are perhaps the most extreme case of mechanistic representation, in which collections of thousands of metabolic reactions are extracted from the genomes of living organisms and represented. While in animal biosciences we refer to such representations as mechanistic, in other fields such models are also referred to as “process-based models” (**PBM**) (Cuddington et al. 2013; Giltrap et al. 2020).

Across disciplines, although MM are useful in quantifying the relationships governing a system, they are limited by the current state of knowledge within the field and the availability of appropriate experimental data to derive their equations. As a result, the performance of such a model can be intrinsically limited by what we know or believe to be true, and requires continual updating as knowledge advances and as our focus and interest evolves. Furthermore, MM can experience problems as they increasingly attempt to integrate processes across multiple temporal and spatial scales (e.g. animal ↔ herd ↔ farm; or fast metabolic processes ↔ slower growth and management responses), introducing well-known tensions around data resolution, subsystem coupling, computational burden, and validation across scales (Neumann et al. 2011; Baker et al. 2018; Jacobs et al. 2022; Reed et al. 2025; Tedeschi et al. 2025). Nevertheless, MM are widely used in agriculture, biology, and related disciplines as they provide a cost-effective supplement to expensive experiments, offer a way to explore systems where experimental studies may be impractical or ethically constrained, and provide a platform to explore system regulation hypothesis (Baker et al. 2018). In practice, such models let us examine “what if” scenarios, thereby providing valuable low-risk decision support and opportunity analysis in the field (Ferguson 2015).

In contrast, AI is not constrained by predefined mechanistic structures derived from scientific first principles, and are data-driven rather than knowledge-driven (Miedema et al. 2026). Sheikh et al. (2023) describes AI as a system-level technology capable of performing diverse tasks that have previously required human intelligence. As such, AI represents a broad umbrella term encompassing multiple computational paradigms, including ML, deep learning (**DL**), reinforcement learning, computer vision, data mining, natural language processing, etc. (Xu et al. 2021; Hassoun et al. 2022). Focusing on ML, these data-driven approaches rely on algorithms, such as Neural Networks, Random Forests, Support Vector Machines, and Gaussian Processes, to map patterns and formulate relationships between inputs and outputs. They are well-suited to handle complex high-dimensional datasets where causality and correlations might not be apparent or fully understood, and where accurate prediction is typically the desired outcome. These ML models excel at capturing multivariable linear and nonlinear patterns, which can achieve high predictive accuracy and support data-driven decisions in real-world production environments with variable conditions. In agricultural applications, ML has been used for a wide variety of tasks from predicting crop yield, to disease onset, to feed intake in livestock (Moshou et al. 2014; Hyde et al. 2020; Morales and Villalobos 2023; Koşkan et al. 2025).

Although ML models are highly adaptable, handle large datasets well, and are useful for recognizing patterns in the data they are given, they also come with limitations. One of the most discussed limitations to consider is that ML models are frequently developed for and evaluated primarily on predictive performance, while the internal structure and decision mechanisms of the trained models are rarely reported in a form that allows scientific interpretation or even replication (Petch et al. 2022; Sheikh et al. 2023). As a result, many modern AI algorithms are applied and reported on as “black boxes,” limiting mechanistic understanding and hindering reproducibility where key aspects of internal model architecture, parameterization, and even training procedures are insufficiently documented (Hutson 2018; Carvalho et al. 2019; Rudin 2019; Pineau et al. 2021). Furthermore, ML model performance is strongly dependent on data quality and variety, meaning these models are only as robust as the data used to train them (Zhou et al. 2024). This limitation is closely linked to “generalizability,” as models trained on a specific dataset may perform well only within the system or conditions under which they were developed (Maleki et al. 2023). When applied to a new animal, farm, region, or management environment, predictive performance may decline rapidly due to differences in the data that would otherwise alter the AI model’s calibration. Though the latter criticism is true of all models, it is most pronounced when models rely on datasets lacking imposed or controlled contrasts and instead rely on natural variation in observational or commercial datasets, which can lead algorithms to exploit spurious correlations rather than biologically meaningful relationships and therefore limit generalization ability (Geirhos et al. 2020).

The juxtaposition of these paradigms—MM versus ML—has been characterized as a battle between “*big theory vs. big data*,” and has received much attention across disciplines (Succi and Coveney 2019; Tedeschi 2019; Ellis et al. 2020).

### The transition to AI use in animal science

The scientific literature, and perhaps with greater sensitivity, proceedings from prominent animal science modeling conferences, can provide insight into the evolution of the scientific modeling paradigm in the animal biosciences field. From a systematic review of historic conference abstract titles published from the prominent animal science modeling conferences: 1) Modeling Nutrient Utilization in Farm Animals (**MODNUT**), 2) American Society of Animal Science National Animal Nutrition Program Workshop (**ASAS-NANP**), 3) American Dairy Science Association (**ADSA**) Modeling Group Meeting, and the 4) European Association for Animal Production (**EAAP**) AI 4 Animal Science Meeting, a search for abstract title key words including “*artificial intelligence*,” “*machine learning*,” “*deep learning*,” “*machine vision*,” “*classification*,” “*clustering*,” “*dimensionality reduction*,” “*computer vision*,” “*precision*,” “*sensor*,” and other related key words such as “*neural network*,” “*decision tree*,” “*real-time*,” “*automated*,” and “*digital*,” was conducted. The result of this systematic review of conference presentation titles is presented in Table 1, along with links to the sources used for count extraction.

**Table 1:** Systematic review results—survey of animal science modeling conference abstract titles for focus on the field of artificial intelligence.

Conference	Year	Total number abstracts published	Number of abstracts about AI/ML/precision^1^	% about AI	Source
**MODNUT**	2025	75	14	19	https://doi.org/10.1016/S2772-283X(25)00888-X
**MODNUT**	2022	89	11	12	https://doi.org/10.1016/S2772-283X(22)01157-8
**MODNUT**	2019	82	4	5	https://doi.org/10.1017/S2040470019000025
**MODNUT**	2014	60	2	3	https://doi.org/10.1071/ANv54n12abstracts
**MODNUT**	2009	45	1	2	https://doi.org/10.3920/978-90-8686-712-7
**ADSA Modelers Meeting**	2025	.	.	.	(Not published at time of review)
**ADSA Modelers Meeting**	2024	11	3	27	https://doi.org/10.1139/cjas-2024-0152
**ADSA Modelers Meeting**	2023	9	2	22	https://doi.org/10.1139/cjas-2024-0024
**ADSA Modelers Meeting**	2021	11	1	9	https://doi.org/10.1139/cjas-2022-0008
**ADSA Modelers Meeting**	2020	6	0	0	https://doi.org/10.1139/cjas-2020-0114
**ADSA Modelers Meeting**	2018	16	0	0	https://doi.org/10.1139/cjas-2018-0140
**NANP-ASAS-CSAS**	2025	6	4	67	https://animalnutrition.org/workshops-symposia
**NANP-ASAS-CSAS**	2024	6	5	83	https://animalnutrition.org/workshops-symposia
**NANP-ASAS-CSAS**	2023	7	4	57	https://animalnutrition.org/workshops-symposia
**NANP-ASAS-CSAS**	2022	6	3	50	https://animalnutrition.org/workshops-symposia
**NANP-ASAS-CSAS**	2021	9	6	67	https://animalnutrition.org/workshops-symposia
**NANP-ASAS-CSAS**	2020	5	2	40	https://animalnutrition.org/workshops-symposia
**NANP-ASAS-CSAS**	2019	5	1	20	https://animalnutrition.org/workshops-symposia
**NANP-ASAS-CSAS (first)**	2018	8	0	0	https://animalnutrition.org/workshops-symposia
**EAAP—AI 4 Animal Science (first)**	2025	73	73	100	https://eaap.org/event/eaap-conference-artificial-intelligence-4-animal-science/

1 conference proceeding titles and abstracts were searched for the terms ‘artificial intelligence’, ‘machine learning’, ‘deep learning’, ‘machine vision’, ‘classification’, ‘clustering’, ‘dimensionality reduction’, ‘computer vision’, ‘precision’, ‘sensor’, and other related key words such as ‘neural network’, ‘decision tree’, ‘real-time’, ‘automated’, and ‘digital’.

Across the major animal science modeling conferences reviewed, AI-linked terminology has shifted from rare, isolated appearances to a prominent and rapidly expanding theme (Table 1). At MODNUT, early examples of precision system modeling and AI application appeared in 2009 and 2014, but represented only 2% to 3% of published abstracts. By 2019, discussion had shifted toward the potential for AI to augment mechanistic models in animal nutrition, and by 2025, 19% of abstracts included some aspect of AI or hybrid modeling. A similar pattern was observed at the Animal Science Modelling Group meeting hosted at ADSA, where AI-linked words first appeared in 2021 and accounted for 27% of abstracts by 2024. The ASAS-NANP symposium series, launched in 2018 with an emphasis on training and methodological innovation, pivoted rapidly toward AI, with 83% and 67% of abstracts addressing AI-related topics in 2024 and 2025, respectively. The launch of EAAP’s “AI 4 Animal Science” conference in 2025, where all abstracts addressed some application of AI, further illustrate the rapid emergence of AI as a central topic in animal science modeling.

In order to understand how disciplines are simultaneously converging on the next paradigm of how we do science, this review will explore first the evolution of agricultural and scientific paradigms and how we arrived where we are (in an AI “boom” in animal science research), along with identified limitations, and explore the emergence of new hybrid paradigms that address limitations of past and present paradigms.

## The evolution of agriculture and science

### How we arrived at agriculture 4.0

Agriculture as a field has undergone a number of significant revolutions, generally following the Industrial Revolutions, that have drastically shaped the face of crop and animal farming systems. To understand “Agriculture 4.0,” one should look to the Agricultural Revolutions that came before it. The first Agricultural Revolution (“*Agriculture 1.0*”), also referred to as the Neolithic Revolution, occurred around 5,000 to 10,000 BCE, with the transition from hunting and gathering to settled agriculture (Weisdorf 2005). In it we saw the domestication of plants into crops (like wheat and barley), as well as the domestication of animals (dogs, sheep, goats, cows, pigs) (Zeller and Göttert 2019). The second Agricultural Revolution (“*Agriculture 2.0*”), also referred to as the British Agricultural Revolution (17th to 19th century), saw innovations such as crop rotation, advancements of ploughs, development of a national market, transportation infrastructure (roads, canals), increase in farm size and selective breeding (Martin 2023). These advancements led to unprecedented increases in land productivity in Great Britian, and the reduced agricultural workforce added to the urban workforce, on which industrialization depended. The third Agricultural Revolution (“*Agriculture 3.0*”) occurred around the 1950s to 1960s, is also referred to as the “Green Revolution,” and saw the adoption of new technologies that increased productivity: high-yield varieties of cereals, genetically modified crop strains (**GMOs**), fertilizers, pesticides, agrochemicals, irrigation, mechanization, and the use of computers (Martin 2023). This agricultural revolution led to widespread reduction in poverty, averted hunger for millions, raised incomes, reduced greenhouse gas (**GHG**) emissions, and reduced land use for agriculture (Pingali 2012). Finally, the 4th Agricultural Revolution (“*Agriculture 4.0*”), which is occurring presently (though there is no consensus yet on its exact start year), has taken shape based on the gradual development of a fundamental tripod: Internet of Things (**IoT**), AI, and ubiquitous connectivity to cloud computing (Albiero et al. 2020). Via Agriculture 4.0, we are increasingly seeing the implementation of emerging digital technologies, AI, big data, sensors, and robotics in agriculture, with the potential to drastically change the face of farming (Albiero et al. 2020; Das and Kamal 2025). The full impact of Agriculture 4.0 may still be considered to be in its infancy, and is yet to be fully realized, but what we are seeing is a gradual shift from the human being in the loop for decision-making and action, to on the loop for supervision, and eventually toward out of the loop complete automation of decisions and/or tasks (Holzinger et al. 2024).

### The four paradigms of science

Just as agriculture as a field has gone through a number of revolutions, the field of Science has evolved through four major paradigms of scientific inquiry as well, being broadly defined as: empirical, theoretical, computational, and data-intensive, as illustrated in Figure 1 (Hey et al. 2009). A paradigm can be broadly defined in science and philosophy as a distinct set of concepts or thought patterns, including theories, research methods, and standards, for what constitutes legitimate contributions to a field across a period of time. Kuhn described a paradigm as “*what the members of a scientific community share, and conversely, a scientific community consists of [a group of people] who share a paradigm*” (Kuhn 1962). As such, the agricultural revolutions described above can also be referred to as socio-technical paradigms within agriculture.

**Figure 1:**
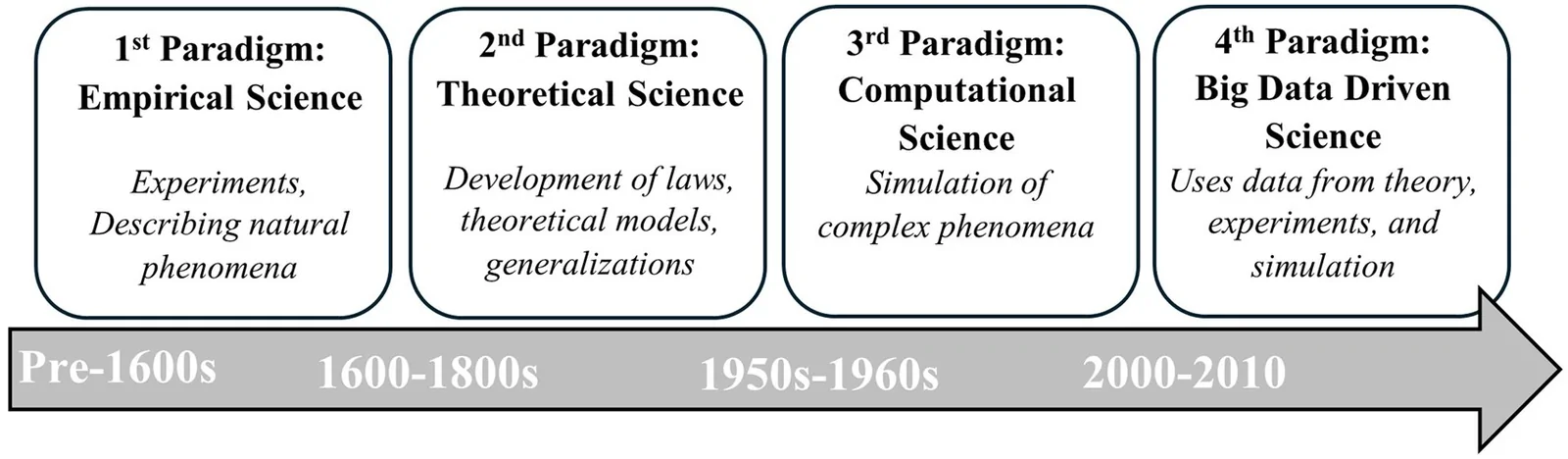
The four scientific paradigms and their approximate start periods (Hey et al. 2009).

Within science, early research was predominantly empirical, relying on observation of natural phenomena, experimentation, and experiential knowledge. The emergence of theoretical science, particularly from the 17th century onward, introduced formal laws and equations to describe the biological, chemical, and physical processes that underpin observations. From the mid-20th century, advances in computing enabled a computational paradigm, in which mechanistic simulation models were developed and used to explore complex, nonlinear systems beyond analytical tractability. More recently, a data-intensive paradigm has emerged, driven by sensors, high-throughput measurements, big-data and AI methods that enable pattern discovery at unprecedented scales.

Consistent with The Structure of Scientific Revolutions (Kuhn 1962), these paradigms continue to coexist, rather than replace one another completely, though a shift in dominance is generally evident. The shifting dominance of scientific paradigms can also be aligned with major agricultural revolutions and paradigm shifts—from traditional husbandry, through industrialized and Green Revolution agriculture, to contemporary digital and sustainability-focused systems. This framing highlights agriculture as a paradigmatic socio-technical science, characterized by periodic redefinition of problems, tools, and criteria for success.

### Paradigm shift

Paradigm shift theory (Figure 2) was first conceptualized by Thomas Kuhn in The Structure of Scientific Revolutions (Kuhn 1962), and provides a new perspective on the nature of the progression of knowledge.

**Figure 2:**
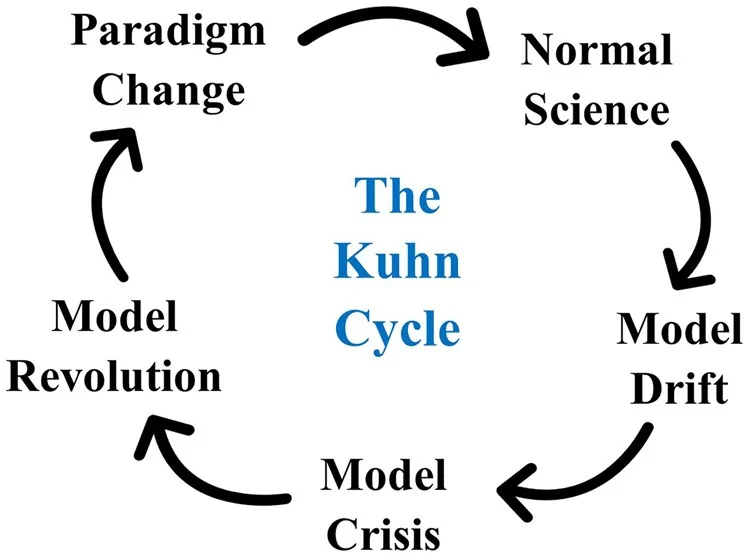
The Kuhn (1962) cycle of paradigm shift.

The first stage of a paradigm shift cycle is what Kuhn describes as “*normal science*,” which involves a period of unproblematic problem-solving of well-defined questions, using widely accepted methodology and a shared vocabulary (Veraart and Blok 2025). This is a period that best describes what Kuhn defines as a (scientific) paradigm.

Normal science begins to erode when faced with *anomalies*, or exceptions that the dominant theory cannot explain. Not every anomaly leads to a paradigm shift, and often prevailing paradigms resist change, defending their truth claims as long as possible (Veraart and Blok 2025), but begin to strain when anomalies accumulate. This strain period is commonly referred to as the second, “*model drift*,” phase.

When the paradigm’s core concepts no longer function effectively and instability deepens, the third stage of paradigm shift, the “*model crisis*,” is entered. At this point, there is too much that the current model cannot explain, the field is thrown into crisis, and a rapid search for a new model begins.

The fourth stage, the “*exploration of alternative theories*,” or “*model revolution*,” emerges from the crisis stage. New models appear, evolve, and compete to explain the anomalies. The old paradigm gradually decays as new groups and schools with different approaches emerge, one of which will ultimately become the new dominant paradigm. When the “*paradigm change*” stage occurs it typically experiences resistance and often still takes years, decades, or even a generation to occur. The cycle is complete when a new paradigm is accepted by most members of the field and taught to younger scientists. At this point, the paradigm change is complete.

### Agricultural socio-technical paradigm shifts

The work by Kuhn has inspired research and reflection on progress throughout various disciplines, well beyond the field of science knowledge advancement (for example, sociology—Given 2008; Steyn 2022; political theory—Foucault 1970; Pirozelli 2021). In addition, Kuhnian paradigm shift theory has been applied not only to scientific world views and grand scientific models or belief systems, but also to the transformation of systems, such as the contemporary digital transformation of agri-food systems (Veraart and Blok 2025). As such, the agricultural revolutions discussed above may also be interpreted as socio-technical paradigms, where each revolution involved a coherent worldview about production, resource use, labor, and what counts as “efficiency” or progress, and Kuhnian paradigm shift theory may be applied to describe the transition between socio-technical paradigms and fundamental shifts in dominant assumptions, tools and criteria for success within socio-technical production systems.

For example, in the preindustrial period (Agriculture 1.0), the dominant paradigm in agriculture was land-labor balance and low external inputs. In the second agricultural revolution (Agriculture 2.0), the dominant paradigm became crop rotation, selective breeding and mechanization. In the green revolution (Agriculture 3.0) the paradigm shifted toward yield maximization via genetics, fertilizer, irrigation, and animal nutrition. Finally, in Agriculture 4.0, the paradigm pivoted to data-driven optimization and systems integration due to the development of disruptive technology (AI, big data, connectivity). Each transition reframed what the problem was in agriculture, e.g. food scarcity → yield per hectare → efficiency per unit environmental cost → digital system optimization.

Examining the ongoing paradigm shift from Agriculture 3.0 to Agriculture 4.0, Agriculture 3.0, or the green revolution, might be considered a phase of “normal science,” with a productionist paradigm prevailing with yield and efficiency as primary goals within agriculture. Within the agricultural scientific community, experimental design and controlled studies are the gold standard, and research is “puzzle-solving” focused on how to increase yields. Anomalies not solved by the yield maximization paradigm and leading to the model crisis phase included emerging problems such as environmental degradation, global warming, and unsustainable input use (Hansen et al. 2000; Tilman et al. 2002), as well as concerns about healthiness of fertilizers, GMO’s, pesticides, hormones and antibiotics (Pretty and Bharucha 2015; Van Boeckel et al. 2015). Initially, according to Kuhnian theory, the main paradigm was not replaced, but rather attempts were made to adapt it by adding rules such as laws, regulations, and policies that steer the food system to achieve its expected outcomes (Veraart and Blok 2025). However, soon, new models began to appear (model revolution), including system paradigms focused on sustainability, resilience, and one health. Although the sustainability paradigm has emerged as a response to the crisis of the Green Revolution, it never fully consolidated as a dominant Kuhnian paradigm, instead, its normative constraints were rapidly absorbed into an optimization paradigm that preserved yield-centric goals while redefining the tools of agricultural decision-making. The digital optimization paradigm that has emerged, following on the heels of disruptive digital technology in Industry 4.0, that has promised win-win solutions for agriculture (efficiency + profitability), fits with existing economic and industrial structures, and it provides new methods (“how do we optimize inputs, timing, and decisions under uncertainty?”) for optimization with the ability to integrate sustainability goals and production constraints. Figure 3 provides a depiction of the Kuhnian paradigm shift cycle applied to the shift between Agriculture 3.0 and 4.0.

**Figure 3:**
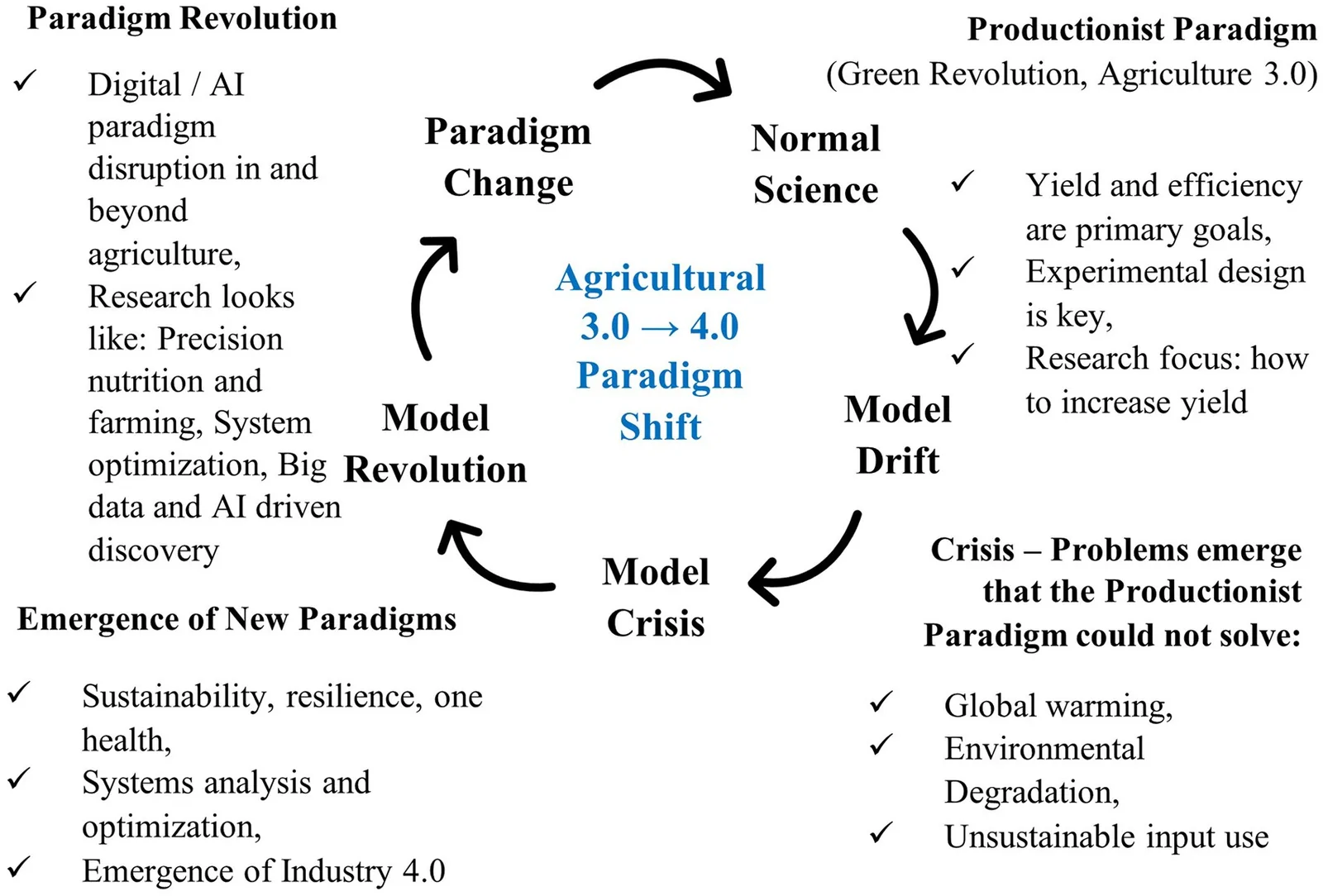
The Kuhnian cycle of paradigm shift applied to the shift from the third to fourth agricultural revolutions.

However, it is interesting to note that the digital paradigm of Agriculture 4.0 has not fully replaced either of the other paradigms (productionist paradigm, transitional sustainability paradigm). The productionist paradigm persists in the industry, the systems paradigm dominates in sustainability, and the digital optimization paradigm is emerging as the “disruptive revolution” layer, driven not by better models to describe anomalies but by technological advancement. Even still, the relatively slow uptake of digital practices in agriculture shows that traditional, “nonsmart” systems are still a major part of everyday work (Cisternas et al. 2020; McFadden et al. 2022; Veraart and Blok 2025). The slow uptake of new digital technology and paradigms may also reflect the risk-sensitive nature of agricultural decision-making, where slower adoption of novel technologies is shaped by uncertainty about return on investment, compatibility with existing production systems, farm-level capacity, trust, and perceived risk (Pannell et al. 2006; Dibbern et al. 2024). Therefore, it might be postulated that agriculture still exists in a state of “paradigm turbulence,” where multiple paradigms coexist, and the transition to a unified next paradigm is not necessarily complete (Veraart and Blok 2025).

### The emerging fifth paradigm

A recent paper by Succi and Coveney (2019) is tauntingly entitled “*Big data: the end of the scientific method?*” They ponder whether the power of “more and more data” renders the scientific method obsolete (*model crisis*/*model revolution*), which is indeed a question often asked within the fourth scientific paradigm and within Agriculture 4.0. For if anything can be inferred from detecting patterns in bigger and bigger data, what is the point of executing the self-reinforcing induction-deduction loop between experimental data generation and theoretical analysis? Authors such as Halevy et al. (2009) argue that when data is sufficiently large, even simple ML models will outperform elegant theory-driven models, in particular in pattern-rich domains such as language and visual data. However, Succi and Coveney (2019) write “*We argue that the boldest claims of big data are in need of revision and toning-down, in view of a few basic lessons learned from the science of complex systems.*” Emerging limitations raised around big data-driven research focus around several key issues: 1) complex systems are strongly correlated, 2) no data is large enough for systems with strong sensitivity to data inaccuracies, 3) correlation does not imply causation, and the link between the two becomes exponentially fainter as data size increases, and 4) in a finite-capacity world, too much data might be just as problematic as too little, leading to saturation, diminishing returns, or even conflicting information that data accumulation alone cannot resolve. Succi and Coveney (2019) state “*In science, we strive to go from data-starved to data-rich, yet a blind data-driven procedure, as often advocated by the most enthusiastic big data neophytes, may well take us from data-rich to data-buried science, unless a just dose of theoretical reasoning is used as an antidote.*”

In response to these limitations, there is increasingly recognized discussion of the emergence of a fifth paradigm of scientific discovery, characterized by the integration of AI with mechanistic theory, autonomous experimentation and causal inference (Xu et al. 2021). Succi and Coveney (2019) state “*once the most extravagant claims of big data are properly discarded, a synergistic merging of big data with big theory offers considerable potential to spawn a new scientific paradigm capable of overcoming some of the major barriers confronted by the modern scientific method*” (Succi and Coveney 2019).

In this new paradigm, AI will automate many research tasks, accelerate the pace of discovery and allow scientists to focus on more complex challenges. The use of AI to process, analyze, and interpret data will enable new levels of discovery and innovation, and will be used to generate new hypotheses from massive datasets (Wang et al. 2023; OpenAI 2026). However, beyond simply collecting massive amounts of data, the movement will be toward understanding and leveraging data via sophisticated computational tools. Unlike the data-intensive fourth paradigm of science, this intelligence-driven fifth paradigm emphasizes the coevolution of models, data, and hypotheses, aligning closely with Kuhn’s concept of paradigm reorganization in response to accumulating anomalies (Kuhn 1962). The recognized anomalies of the fourth paradigm (big data driven science) include: poor extrapolation outside training domains, limited causal interpretability, and weak biological plausibility. The fifth paradigm emerges as a reorganization, rather than an extension: models are constrained by mechanistic structure, AI assists in model discovery, refinement, validation, generalization, and explanation regains primacy alongside prediction (Baker et al. 2018; Wang et al. 2023).

It is from such discussions that model hybridization, or the deliberate combination of mechanistic knowledge with AI, emerges across disciplines.

## Model hybridization

Across disciplines, the use of the term “hybrid modeling” is not well agreed upon. In some papers, the term is used to refer to modified structures applied to pure nonparametric approaches for prediction, such as an artificial neural network (**ANN**) implemented with other ML techniques or optimization algorithms (Park et al. 2002; Kilinc 2022), ensemble models composed from a number of ML models (Asad et al. 2023; Madhuvanthi and Baskaran 2025), or even student-teacher frameworks (Hinton et al. 2015) where a large “teacher” neural network transfers information to a smaller “student” neural network. Additionally, one might consider “grey-box” modeling (combining empirical and mechanistic components) a hybrid approach (Hauschild et al. 2012; Ye et al. 2015; Jacobs et al. 2022; Railsback et al. 2025).

Herein, hybrid modeling refers to the intentional integration of mechanistic (theory-driven) models with data-driven or ML components within a single modeling framework, such that each paradigm compensates for the limitations of the other. As such, hybrid modeling can be defined as the integration of mechanistic, theory-based representations of biological or physical processes with data-driven or ML components. The resulting hybrid model generally shows enhanced predictive performance (compared to MM alone) and improved interpretability (compared to ML alone) (demonstrated in examples provided below), and aligns with the emerging fifth paradigm of AI-augmented scientific discovery.

Hybrid modeling, defined in this manner, has been adopted as early as the 1990s in fields such as bioprocess engineering (Psichogios and Ungar 1992; Agarwal 1997) (1992 being one of the earliest formal definitions of hybrid modeling), physics (Karniadakis et al. 2021), systems biology (Procopio et al. 2023; Noordijk et al. 2024), chemical engineering (Venkatasubramanian 2019), climate science (Slater et al. 2023), and human health research (Wong et al. 2024), and a number of useful reviews of the topic across these other field have been produced (e.g. Herrera‐Ruiz et al. 2025).

Hybrid approaches have been shown to be particularly useful for uncovering relationships that may be difficult to capture with traditional modeling strategies that rely on a single framework, such as purely mechanistic or purely data-driven models. By combining MM/PBMs with flexible, data-driven learning, hybrid models can improve predictive accuracy in highly variable environments while still preserving a structured, scientifically grounded foundation (Rudolph et al. 2024; Herrera‐Ruiz et al. 2025). As industrial datasets continue to expand and big data becomes increasingly accessible, hybrid models represent a new frontier in decision-making, helping accelerate development in bioprocesses and related applications (Gargalo et al. 2024; Herrera‐Ruiz et al. 2025). Figure 4 provides a visual representation of data-driven, mechanistic, and hybrid modeling, along with the types of knowledge (process data vs *a priori* knowledge) they utilize.

**Figure 4:**
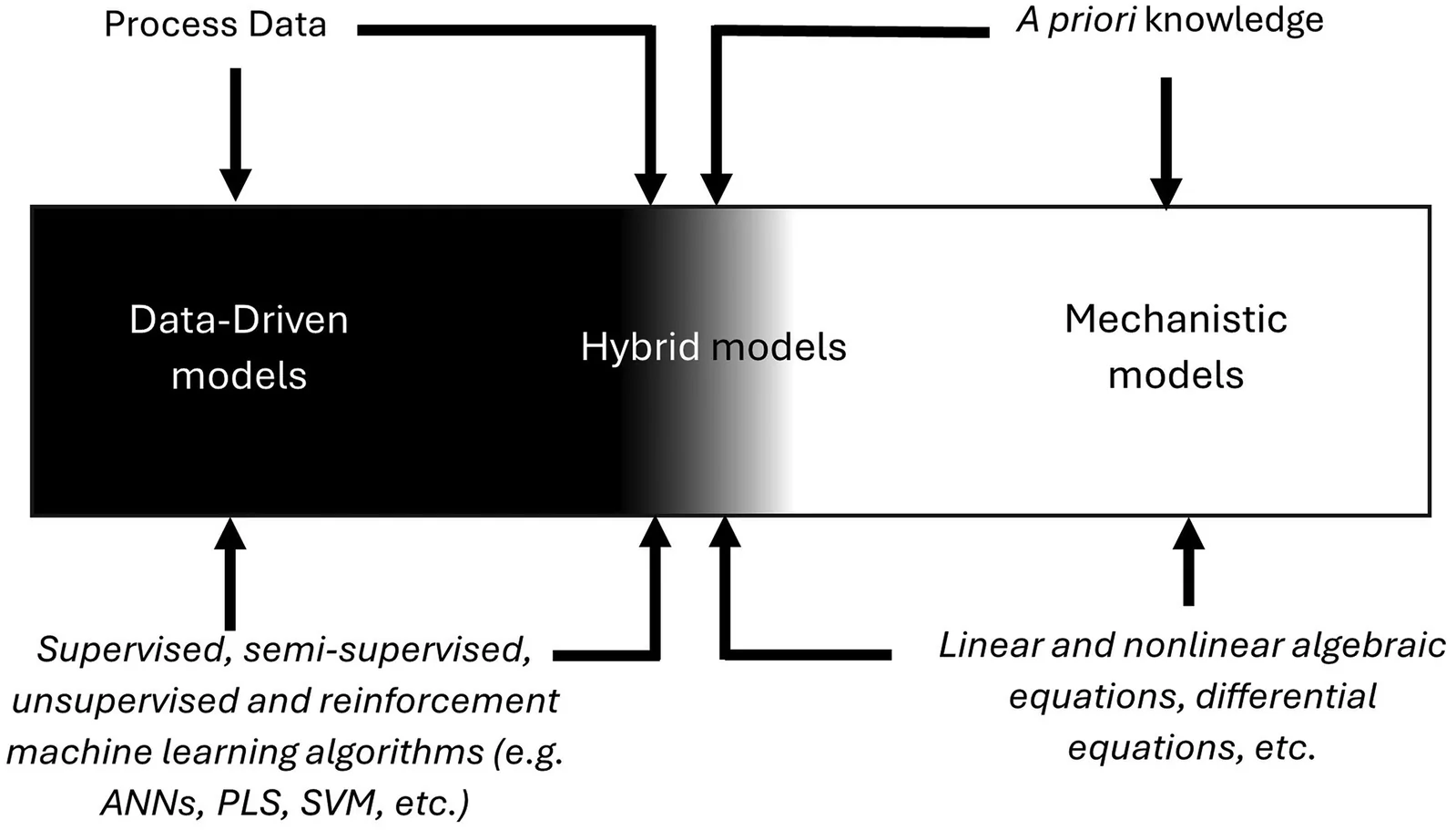
Data-driven, mechanistic and hybrid modeling and the type of knowledge they are based on. Adapted from Herrera-Ruiz et al. (2025).

### Hybrid model architectures

A number of hybridization design architectures have been proposed, reviewed, and implemented for hybrid modeling applications across disciplines, as nicely synthesized by Rudolph et al. (2024), and five common architectures are presented in Figure 5. Such architectures provide the basic building plan for the development of hybrid models, where each pattern consists of two computational models, a first-principles-based MM and a data-driven AI model, and combines their computational steps into a hybrid model. Each architecture differs in the order in which the computation is executed and in the flow of inputs and outputs between the components. The five basic hybrid model design structures (Figure 5) are detailed below.

**Figure 5:**
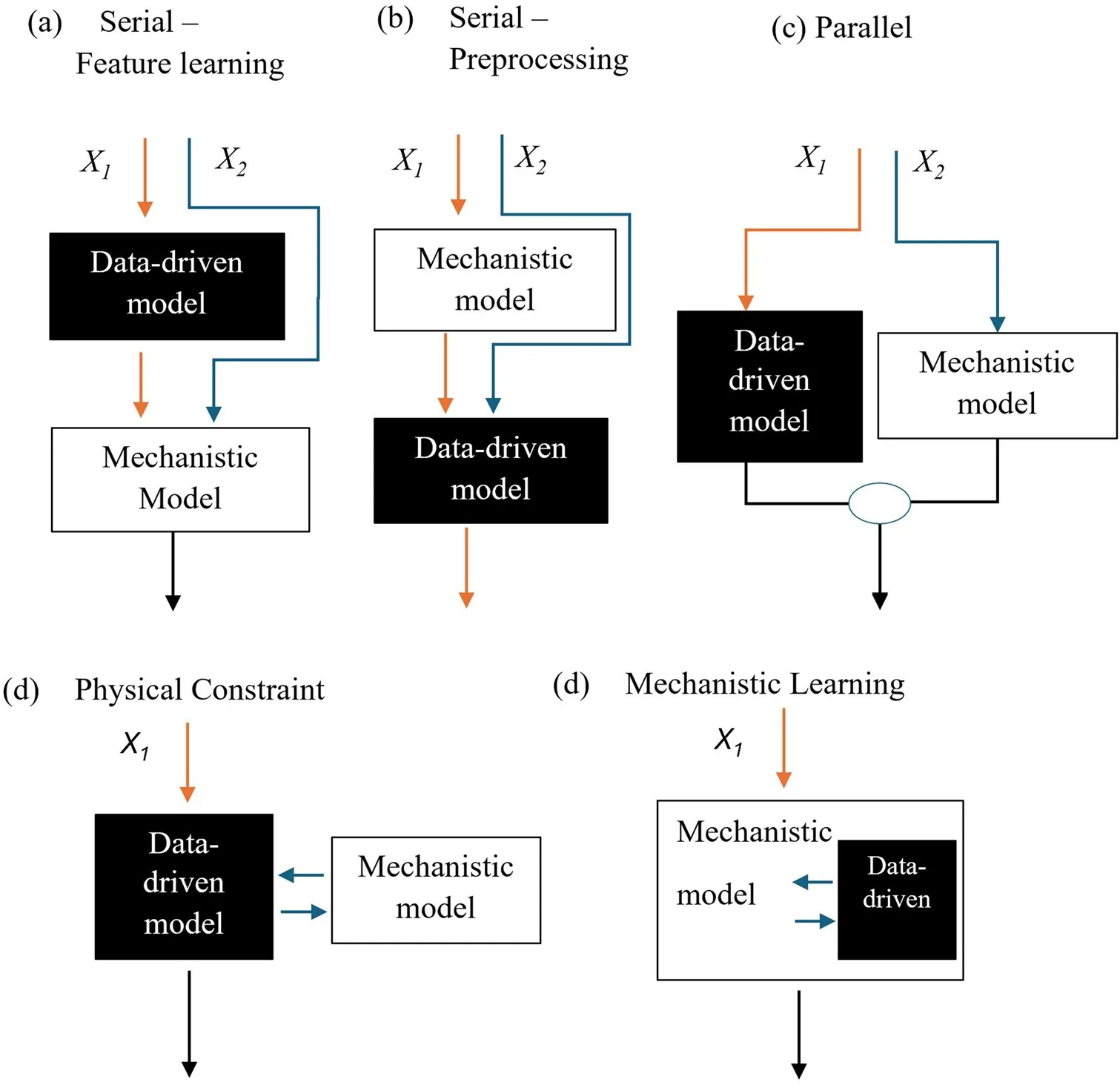
Examples of architecture design patterns for building hybrid models: a) serial hybridization–feature learning, b) serial hybridization–preprocessing, c) parallel hybridization, d) physical constraint hybridization, and e) mechanistic learning hybridization. Adapted and expanded from Herrera-Ruiz et al. (2025) and Agarwal (1997).

#### Serial hybridization

Serial model hybridization refers to a model-building strategy in which distinct models are linked sequentially, such that the output of one model becomes the input, constraint, or residual target of the subsequent model (Figure 5a and b). When the data-driven model is first, followed by a MM (Figure 5a), this is referred to as a “*feature learning*” serial hybridization. When the MM is first (Figure 5b), this is referred to as “*physics-based preprocessing*” serial hybridization. The feature learning approach has been applied where knowledge and a general valid model to describe a process or transport kinetics is absent (for example, as needed for a mechanistic model), while the preprocessing approach is predominantly used in data-sparse environments (Rudolph et al. 2024; Herrera‐Ruiz et al. 2025).

A special case of physics-based preprocessing is “*surrogate*” modeling, in which outputs generated by a MM are used to train a data-driven ML model that approximates the MM response. In this configuration, hybridization occurs during model development, as the MM provides simulated training data, while during prediction the surrogate replaces the MM to provide computationally efficient approximations. As such, surrogate modeling represents the lowest level of mechanistic integration within hybrid architectures, as mechanistic structures inform model training but the MM is not utilized directly during prediction. Surrogate approaches are commonly used to accelerate computationally intensive simulations, enable optimization or uncertainty analysis, or facilitate real-time prediction while retaining information learned from mechanistic simulations (Forrester and Keane 2009).

When using serial architectures, model training can be performed via: 1) coupling the parameterization and fitting of the two models (simultaneous) or 2) decoupling the model parameterization and fitting by calibrating the mechanistic model first, and then the data-driven model to account for the deficiencies in the MM (Herrera‐Ruiz et al. 2025), for example.

#### Parallel hybridization

Parallel model hybridization is a modeling strategy in which mechanistic and data-driven models are developed and executed independently on the same system, and their outputs are subsequently combined through an explicit integration layer to generate final predictions (Figure 5c). This form of hybridization is also referred to as a “*delta model*” design architecture and is typically applied when there is a mechanistic model that captures the underlying mechanisms but has limited predictive power due to variables not accounted for in its structure such as unmodeled effects, nonlinearities, or dynamic behavior (Rudolph et al. 2024; Herrera‐Ruiz et al. 2025). The data-driven model would account for these components.

Inputs to the data-driven and MM components under this design architecture could be the same or different (for example, in the latter case, the data-driven component might obtain knowledge from information the MM does not consider), and there are numerous options for integrating the predictions from the two models. “*Superposition*” is where the data-driven model output is summed or subtracted from the MM output (the most commonly applied parallel architecture), “*multiplication*” is where the data-driven output is multiplied by the MM output (serving as a correction factor), and “*transformation*” is where the output of the data-driven and MM are further transformed by either an additional data-driven or MM layer (Rudolph et al. 2024; Herrera‐Ruiz et al. 2025).

#### Physical constraint hybridization

Physical constraint hybridization is an approach in which mechanistic laws, biological principles, or conservation constraints are explicitly embedded within data-driven models, restricting the space of admissible solutions to those that are physically or biologically plausible (Figure 5d). Rather than coupling separate models sequentially or in parallel, this strategy integrates constraints directly into the learning process, shaping model structure, parameter ranges, loss functions, or feasible outputs (Rudolph et al. 2024; Herrera‐Ruiz et al. 2025). The defining feature of physical constraint hybridization is that learning is guided—not corrected—by biology. This distinguishes it from serial hybridization (where learning targets residuals) and parallel hybridization (where models are combined post hoc). By embedding first principles directly into the model, this approach aims to improve interpretability, stability, and extrapolation, particularly under novel scenarios, environments or genetics within a single model. Mathematically there is also an alternate configuration to physical constraint hybridization (Figure 5d) that could be considered, Data-driven constraint, where the data-driven and MM models swap positions and the MM is “guided” by a data-driven model.

#### Mechanistic learning

Lastly, mechanistic learning, also referred to as *intrinsic* hybridization or ML-embedded MM (**MLeMM**) (Figure 5e), describes an approach in which ML components operate within the structure of an MM to learn specific processes, parameters, or functional relationships while preserving the governing mechanistic framework (Metzcar et al. 2024). Rather than functioning before or alongside the MM, ML components are embedded internally and interact with other model equations through the same system interconnections as in the original mechanistic formulation. Outputs from embedded ML components may be constrained or bounded to ensure consistency with biological or physical realism.

### Examples of model hybridization from bioprocess engineering

Hybrid models have gained popularity across disciplines as they combine complementary modeling approaches, reducing the limitations of individual methods while leveraging their respective strengths. One such field that has taken advantage of this unique modeling style is bioprocess engineering. Bioprocess engineering refers to the design of industrial-level systems that integrate biotechnology production systems, which can use elements such as microorganisms and cells, to manufacture valuable products (Duong-Trung et al. 2023). The use of model-based decision making in these systems has been quickly adopted as it opens a path to understanding alternative approaches in the development of strategies that can reduce uncertainty in the system (Duong-Trung et al. 2023). As a result, hybrid, or semi-parametric, modeling has become a valuable tool in bioprocess engineering, supporting cost-effective model development, reducing experimental burden, and improving model-based decision-making during process development and manufacturing (von Stosch et al. 2014). Since its inception in the early 90s, hybrid modeling for application in bioprocesses has grown by roughly 9% every year (Herrera‐Ruiz et al. 2025).

Hybrid modeling studies conducted in this field have helped discern information from complex systems with internal systems that are not well known but have large datasets associated with them. For instance, von Stosch et al. (2012) used a hybrid modeling approach (mechanistic learning) to determine metabolite concentrations produced by the bacteria *Bordetella perussis*. The serial semiparametric hybrid model combined first principles information, established through macroscopic material balances and biological fluxes, with the partial least squares (**PLS**) data-driven modeling technique to strengthen prediction accuracy and process understanding. The results demonstrated that the hybrid model produced lower validation Mean Squared Error (**MSE**) where the validation MSE ranged from 0.04 to 0.06 as compared to the standalone PLS data-driven modeling technique with a validation MSE ranging from 0.2 to 0.65 (von Stosch et al. 2012).

Similarly, hybrid models have also been used in wastewater treatment to improve process monitoring and determine model-plant mismatch (Galinha et al. 2013; Hasanlou et al. 2015; Ding et al. 2024; Herrera‐Ruiz et al. 2025), and to model intracellular signaling pathways (Lee et al. 2020).

Recently, Faure et al. (2023) introduced a new type of neural-mechanistic hybrid GEMs (physical constraint hybridization), where constraint-based mechanistic models are intertwined with neural network layers, creating several serial-preprocessing frameworks on top of each other. With these hybrid GEMs they showed significant improvements in growth rate predictions of *Escherichia coli* and *Pseudomonas putida* in different media and with phenotype predictions of gene knocked-out *Escherichia coli* mutants, outperforming both the MM flux balance and ANN approaches. This hybrid method saved time and resources by reducing the need for many additional experimental measurements.

These studies highlight how hybrid modeling can be applied to highly complex biological systems with strong relevance to real-world biological processes and industrial applications.

### Examples of model hybridization from human health research

Hybrid modeling approaches that combine ML with MM are also widely used in human health research to address the computational cost, scalability limits, and data constraints of detailed physiological simulations. Rather than replacing MM, these approaches integrate ML to accelerate computation, support analysis, or embed biological structure while retaining interpretability. Such hybrid strategies have been applied across cardiovascular physiology, pharmacology, immunology, and cancer systems modeling (Alber et al. 2019; Gilpin et al. 2020; Lan et al. 2025).

One common hybrid approach in this field is ML surrogate modeling, where ML models are trained on outputs from mechanistic simulations and then used to reproduce those outputs with the same, or fewer, inputs at substantially lower computational cost (serial hybridization) (Pruett and Hester 2016). In human physiology, surrogate models have been developed for cardiovascular dynamics, coagulation processes, and whole-body physiological systems, achieving processing speed gains of several orders of magnitude while maintaining accuracy for clinically relevant outputs (Pruett and Hester 2016; Bouchnita et al. 2022; Führer et al. 2024). These surrogates enable large-scale simulation, rapid evaluation of treatment scenarios, and patient-specific analyses that would be impractical using full MM alone.

Another hybrid strategy applied in human health uses ML to study the behavior of MM themselves rather than replacing them. In this framework, MM generates large simulated datasets representing virtual patient populations, and ML is then applied for parameter estimation, sensitivity analysis, or outcome prediction (a serial hybridization approach). This approach has been used in studies of immune signaling, cancer therapy response, and pharmacological systems to identify influential parameters and explain variability in patient outcomes (Hua et al. 2006; Zhang et al. 2019; Cess and Finley 2020; Zhang and Tyson 2022).

More tightly coupled hybrid approaches integrate mechanistic knowledge directly into ML workflows. In one strategy, mechanistic models are used for data augmentation and feature engineering, particularly when empirical clinical datasets are small (serial hybridization). By generating biologically informed synthetic data or mechanistic descriptors, these serial hybridization examples demonstrate improved predictive accuracy and model interpretability in applications such as tumor growth modeling and medical imaging (Deist et al. 2019; Gaw et al. 2019; Benzekry et al. 2024).

Other hybrid approaches employed in human health research embed biological or physical constraints directly into ML architectures (physics-informed hybridization). By constraining the hypothesis space, these models improve generalization, stability, and biological consistency in physiological and disease modeling tasks (Raissi et al. 2019; Elmarakeby et al. 2021; Hao et al. 2022; Stapor et al. 2022).

### Examples of model hybridization from weather/environment modeling

Making accurate weather forecasts has long been challenging due to the highly variable and complex nature of Earth’s atmosphere. As meteorological conditions are constantly changing and are influenced by many interacting factors, such as isolated thunderstorms in local geographies, data-centric and hybrid approaches can be valuable tools for uncovering the complex underlying relationships that arise from dynamic environments (Grover et al. 2015).

A deeper look into the hybrid modeling approaches that have been used in weather forecasting demonstrates the significant impact that these models have on improving prediction accuracy. For instance, Wang et al. (2022) describe how although traditional numerical models are beneficial for weather and pollutant forecasting and assessment, their predictions approximate many complex interacting physical and chemical processes that are not fully understood, leading to low-accuracy predictions. To address this limitation, Wang et al. (2022) proposed two hybrid models (serial hybridization—preprocessing) to improve and correct the model’s forecasting residuals, improving predictions of six different pollutant concentrations.

Similarly, Mohamed et al. (2025) developed a hybrid weather and agriculture forecasting model that combined a data-driven AI model (Convolutional Neural Network and Recurrent Neural Network) with rule-based architecture to facilitate decision making (Physics-Informed hybridization). The results indicate that the hybrid models accurately determined weather patterns and provided reliable forecasts for different meteorological variables, as shown by the decreasing MSE and MAE trends during training and their overall stabilization as the model converged (Mohamed et al. 2025).

Other studies have looked into the use of hybrid models in forecasting wind power (Chang et al. 2024; Wang et al. 2025), predicting greenhouse climates (Xiong and Su 2025), and making climate simulations more realistic (Arcomano et al. 2023). Collectively, these examples from weather and environmental modeling illustrate how hybrid approaches can augment numerical and process-based weather models by correcting residual errors, capturing nonlinear relationships, and improving computational efficiency.

### Examples of model hybridization from agriculture

While a number of other fields, discussed above, have seen a steady increase in the development of hybrid models to solve complex system problems, their exploration and use in modeling agriculture systems is still quite novel, with most applications to date having been in agriculture/cropping system as opposed to animal systems.

In one example, Droutsas et al. (2022) used ML within a crop model (serial hybridization–feature learning) to predict radiation-use efficiency as it changes over time and crop development rates under changing environmental conditions, improving simulations of crop growth and yield under heat stress. In another, Dong et al. (2023) used ML to predict root length density changes over time under saline conditions and supplied these estimates to a crop model (serial hybridization–feature learning), replacing a fixed empirical root function and improving crop growth simulations in saline soils.

Feng et al. (2019) used simulated crop-model outputs and climate-extreme indicators as inputs to an ML model (serial hybridization—preprocessing), enabling improved representation of nonlinear wheat yield responses to heat and drought in south-eastern Australia. Shahhosseini et al. (2021) similarly improved corn yield prediction in the U.S. Corn Belt by using simulated state variables from a mechanistic crop model as predictors for an ML algorithm (serial hybridization—preprocessing). At broader scales, Sun et al. (2022) used outputs from a MM together with extreme-climate indicators as inputs to an ML model to reduce uncertainty in soybean yield projections (serial hybridization—preprocessing). Attia et al. (2022) trained ML models on a Decision Support System for Agrotechnology Transfer (DSSAT) simulations to estimate maize yield and evapotranspiration under water-limited conditions (serial hybridization–preprocessing).

In terms of surrogate modeling, Xiao et al. (2022) reported that ML surrogates can accurately and rapidly reproduce outputs from a MM, including yield and soil carbon dynamics at regional scales under a range of crop management practices and scenarios. Similar surrogate approaches have been applied to other agricultural prediction tasks that benefit from rapid evaluation, such as estimating N_2_O emissions from intensively managed cropping systems (Saha et al. 2021). Surrogate modeling is also increasingly applied in computationally intensive farm-system and livestock-environment settings, including DL surrogates of whole-farm models such as FarmDyn (Shang et al. 2024) and ML surrogates for dairy barn ventilation that approximate computational fluid dynamic (**CFD**) outputs while substantially reducing the computational cost of repeated airflow simulations during design and evaluation (Chung et al. 2022).

To date, there are very few published examples of hybrid modeling from animal science, and those that are there are quite recent, and mostly in thesis or conference abstract form (Bode et al. 2025; Fieguth 2025; Kaniyamattam et al. 2025; Sahar et al. 2025; Li et al. 2026).

### Hybrid model benefits, interpretability, and data requirements

Hybrid modeling represents more than a technical compromise between MM and ML; it is a structural reconciliation of complementary epistemologies (Baker et al. 2018; Ellis et al. 2020). Across disciplines, reviewed herein, several consistent benefits emerge: improved predictive performance, enhanced extrapolation capacity, reduced data burden under certain architectures, increased computational efficiency through surrogacy, and improved overall interpretability (Rudolph et al. 2024; Herrera‐Ruiz et al. 2025).

#### Predictive robustness and extrapolation

Mechanistic models provide structural consistency grounded in conservation laws, biological causality, and physiological plausibility (Dijkstra et al. 1992; France and Kebreab 2008). However, they may struggle with nonlinearities, unmodeled interactions, or context-specific deviations. Machine learning models excel at detecting such nonlinear residual structure, but often lack extrapolation capacity beyond their training domain (Maleki et al. 2023). Hybrid architectures—particularly parallel (“delta”) and physically constrained approaches—allow ML components to correct or refine MM predictions while remaining anchored to biological first principles (Rudolph et al. 2024; Herrera‐Ruiz et al. 2025). This reduces the risk of implausible predictions under novel diets, climates, genetics, or management systems.

Importantly, physical constraint hybridization narrows the hypothesis space during learning. By embedding conservation of mass, energy partitioning rules, or biological rate limits directly into the learning objective, the model is prevented from converging on biologically impossible solutions (Raissi et al. 2019; Karniadakis et al. 2021). In livestock systems, where extrapolation to new feeding strategies or environmental scenarios is common, this constraint-driven learning may be particularly valuable (Ellis et al. 2020).

#### Interpretability and explainability

A common critique of ML models is opacity (Petch et al. 2022). However, interpretability tools such as permutation feature importance, SHAP (SHapley Additive exPlanations) values, and partial dependence plots allow quantification of feature importance and nonlinear response relationships (Lundberg and Lee 2017; Tulpan et al. 2026). When deployed within hybrid systems, these tools serve not only diagnostic purposes but hypothesis-generating roles. For example, feature attribution methods can reveal nonlinear dietary responses, interactions between environmental stressors and intake behavior, or threshold effects not previously encoded in mechanistic formulations (Ellis et al. 2020).

Hybrid models thus move beyond “black-box prediction” toward biologically guided pattern discovery. Mechanistic structure provides causal scaffolding while ML identifies where that structure may require refinement (Baker et al. 2018; Tedeschi 2019). In this sense, hybrid modeling restores explanation alongside prediction, addressing one of the core anomalies of the purely data-intensive fourth paradigm (Succi and Coveney 2019).

#### Data requirements: less or different data

A key but often underappreciated benefit of hybrid models is that it may reduce the amount of empirical data required to achieve reliable prediction. Pure ML approaches require large, diverse, and representative datasets to generalize effectively (Morota et al. 2018; Maleki et al. 2023). In contrast, hybrid models capitalize on existing domain knowledge (France and Kebreab 2008). When mechanistic models provide baseline structure, the ML component often learns only the residual or poorly understood subprocesses (Herrera‐Ruiz et al. 2025). This substantially reduces the dimensionality and volume of data required.

However, hybrid modeling does not eliminate the need for high-quality data; rather, it changes its role. Data shift from defining the entire functional relationship to refining, calibrating, or augmenting mechanistic understanding (Baker et al. 2018; Succi and Coveney 2019). Consequently, hybrid systems demand well-curated, biologically consistent datasets.

#### Computational efficiency and implementation speed

In computationally intensive applications—such as whole-farm simulations, ordinary differential equation (ODE) systems for rumen fermentation, or barn-scale CFD airflow modeling—ML surrogate models can reduce runtime by orders of magnitude (Pruett and Hester 2016; Chung et al. 2022; Shang et al. 2024). Once trained, surrogates enable rapid scenario evaluation, sensitivity analysis, and optimization that would otherwise be infeasible in real-time decision-support contexts (Herrera‐Ruiz et al. 2025). Furthermore, GPU-accelerated inference of ML models increases speed by orders of magnitude compared to classical CPU-based algorithms.

For Agriculture 4.0 and 5.0 implementations, this speed advantage is critical. Decision-support systems embedded within feeding software, ventilation controllers, or emission monitoring platforms require near-instantaneous predictions (Berckmans 2017; Wolfert et al. 2017). Surrogate-based hybrid models bridge this gap between mechanistic depth and operational feasibility.

Collectively, these benefits suggest that hybrid modeling is not merely a methodological innovation, but a structural adaptation to the scientific and socio-technical demands of Agriculture 4.0 (Veraart and Blok 2025). The rapid expansion of AI in agriculture has raised concerns regarding what has been described as the “shiny object syndrome,” in which emerging technologies are adopted primarily due to novelty rather than demonstrated scientific necessity or theoretical integration (Tedeschi 2022). In the context of livestock modeling, the uncritical pursuit of increasingly complex ML architectures without mechanistic grounding risks prioritizing computational sophistication over biological understanding. Hybrid frameworks, therefore, should not be interpreted as technological escalation, but as structured integration designed to preserve causal logic while enhancing predictive capacity.

## Toward the fifth scientific paradigm

Scientific paradigms have historically evolved from empirical observation to theory-driven science, to computational modeling, and most recently to data-intensive discovery (Hey et al. 2009). The fourth paradigm—characterized by high-throughput data and ML-driven pattern recognition—has transformed agriculture and animal biosciences (Morota et al. 2018). Yet, as discussed earlier, it exhibits identifiable anomalies: limited causal interpretability, poor extrapolation beyond training domains, and vulnerability to data bias (Succi and Coveney 2019; Maleki et al. 2023).

The emerging fifth scientific paradigm represents not an abandonment of data-driven science, but its reorganization. It is characterized by the deliberate integration of AI with mechanistic reasoning, automated experimentation, and causal inference (Karniadakis et al. 2021; Lan et al. 2025). In this paradigm, AI does not replace theory; it augments it.

This shift reframes the often-polarized narrative of “*big data versus big theory*” to “*big data plus big theory*” (Succi and Coveney 2019). Rather than competing paradigms, ML and MM become coevolving components of a unified modeling philosophy. Hybrid models exemplify this synthesis: AI accelerates parameter estimation, detects nonlinear structure, and automates hypothesis testing, while mechanistic frameworks preserve interpretability, causal reasoning, and biological plausibility (Baker et al. 2018; Tedeschi 2019).

Within Kuhn’s framework (Kuhn 1962), the fifth paradigm can be viewed as a response to the accumulated anomalies of the fourth paradigm. Where data-intensive science struggles with generalizability and explanation, hybrid approaches restore structural grounding (Succi and Coveney 2019). Explanation regains primacy alongside prediction.

From a socio-technical perspective, this transition aligns with Agriculture 4.0’s maturation phase (Albiero et al. 2020; Veraart and Blok 2025). The digital paradigm—currently positioned near the peak of inflated expectations—will inevitably encounter limits as purely data-driven systems confront biological complexity. Hybrid modeling offers a pathway toward more stable and productive integration of digital tools within biologically coherent frameworks.

The implications extend beyond methodology to scientific training. As AI becomes integrated into experimental design, model calibration, and hypothesis generation, computational literacy will become foundational (Jacobs et al. 2022; Tedeschi 2022). Coding, data engineering, and ML literacy are likely to become default competencies, taught from an earlier age, for future animal scientists. However, these skills must develop alongside, rather than replace, deep domain expertise.

Thus, hybrid modeling is not only a technical architecture but an epistemological shift. It signals a transition toward intelligence-augmented science in which data, theory, and computation coevolve (Hey et al. 2009; Karniadakis et al. 2021).

## Conclusions

Agriculture 4.0 requires modeling frameworks that are predictive, interpretable, computationally efficient, and biologically grounded. Mechanistic models alone are constrained by incomplete knowledge and computational burden, while purely data-driven approaches risk opacity, weak extrapolation, and limited causal insight. Hybrid modeling offers a principled synthesis by integrating mechanistic structure with ML adaptability, thereby enhancing predictive performance while preserving biological plausibility. Across livestock systems, such models could enable real-time decision support, improved feed efficiency prediction, more robust emission estimation, and scalable scenario analysis without discarding decades of accumulated mechanistic understanding. As animal biosciences moves toward an AI-augmented scientific paradigm, hybrid models are poised to become central tools for research, training, and implementation. The future of livestock modeling does not lie in choosing between theory and data, but in their deliberate integration to build digitally enabled, causally robust, and sustainable production systems.

## Abbreviations

AI: artificial intelligence
ANN: artificial neural network
CFD: computational fluid dynamic
CMAQ: community multiscale air quality
CNN: convolutional neural network
DL: deep learning
DSSAT: decision support system for agrotechnology transfer
GEM: genome-scale metabolic model
LSTM: long short-term memory
ML: machine learning
MLeMM: ML-embedded MM
MM: mechanistic model
PBM: process-based model
PIML: physics-informed machine learning
PINN: physics-informed neural network
WRF: weather research and forecasting
